# When Pancreas Pierces Pleura: An Uncommon Case of Pancreaticopleural Fistula Managed Endoscopically

**DOI:** 10.7759/cureus.107057

**Published:** 2026-04-14

**Authors:** Arman Manjikian, Victoria Diaz, John K Ryan

**Affiliations:** 1 Internal Medicine, MountainView Hospital, Las Vegas, USA; 2 Gastroenterology, Sunrise Graduate Medical Education Consortium, Las Vegas, USA; 3 Gastroenterology and Hepatology, Comprehensive Digestive Institute of Nevada, Las Vegas, USA

**Keywords:** chronic pancreatitis, endoscopic stenting, ercp, mrcp, pancreatic duct disruption, pancreaticopleural fistula, pleural effusion

## Abstract

Pancreaticopleural fistula (PPF) is a rare complication of pancreatitis and typically presents with large recurrent pleural effusions. Herein, we present an unusually severe PPF in a 37-year-old woman with complete left lung atelectasis from a massive pleural effusion. Pleural fluid amylase exceeded 1500 U/L, and endoscopic retrograde cholangiopancreatography (ERCP) confirmed distal pancreatic duct disruption. The patient’s course was complicated by a transdiaphragmatic pseudocyst, polymicrobial empyema, pulmonary abscess, and stent migration, requiring repeat ERCP and multiple drainage procedures. This case highlights the development of a rare thoracic complication, and demonstrates successful endoscopic management.

## Introduction

Pancreaticopleural fistula (PPF) is a rare sequela of pancreatitis reported in less than one percent of patients with pancreatitis [1,2]. It results from pancreatic duct disruption with retroperitoneal or transdiaphragmatic tracking of enzyme-rich secretions into the pleural space [3]. Most reported cases present with large unilateral pleural effusions but without severe thoracic involvement [4]. Alcohol use disorder and recurrent subclinical pancreatitis are the most common risk factors [1,5]. Because respiratory symptoms predominate in over two-thirds of cases, diagnosis is frequently delayed [4,6]. This case is unique due to the combination of complete lung atelectasis, transdiaphragmatic pseudocyst extension, polymicrobial empyema, and stent migration, which were successfully managed endoscopically.

Diagnosis is typically suggested by markedly elevated pleural fluid amylase levels and confirmed with imaging such as magnetic resonance cholangiopancreatography or endoscopic retrograde cholangiopancreatography (ERCP). Management strategies include conservative therapy, endoscopic pancreatic duct stenting, and surgical intervention in refractory cases [3,5].

## Case presentation

A 37-year-old woman with alcohol use disorder presented with one month of progressive shortness of breath, nonproductive cough and wheezing. She denied chest pain, abdominal pain, or prior episodes of pancreatitis. On physical examination, she was tachypneic with markedly diminished breath sounds and dullness to percussion over the entire left hemithorax. Chest radiograph showed complete opacification of the left hemithorax.

Initial laboratory evaluation was notable for severe hypokalemia with potassium at 1.9 mmol/L (reference 3.5-5.0 mmol/L), elevated aspartate aminotransferase at 107 U/L (reference 10-40 U/L), hypoalbuminemia with albumin at 2.1 g/dL (reference 3.5-5.0 g/dL), and mildly elevated lipase at 54 U/L (reference 13-60 U/L).

**Figure 1:**
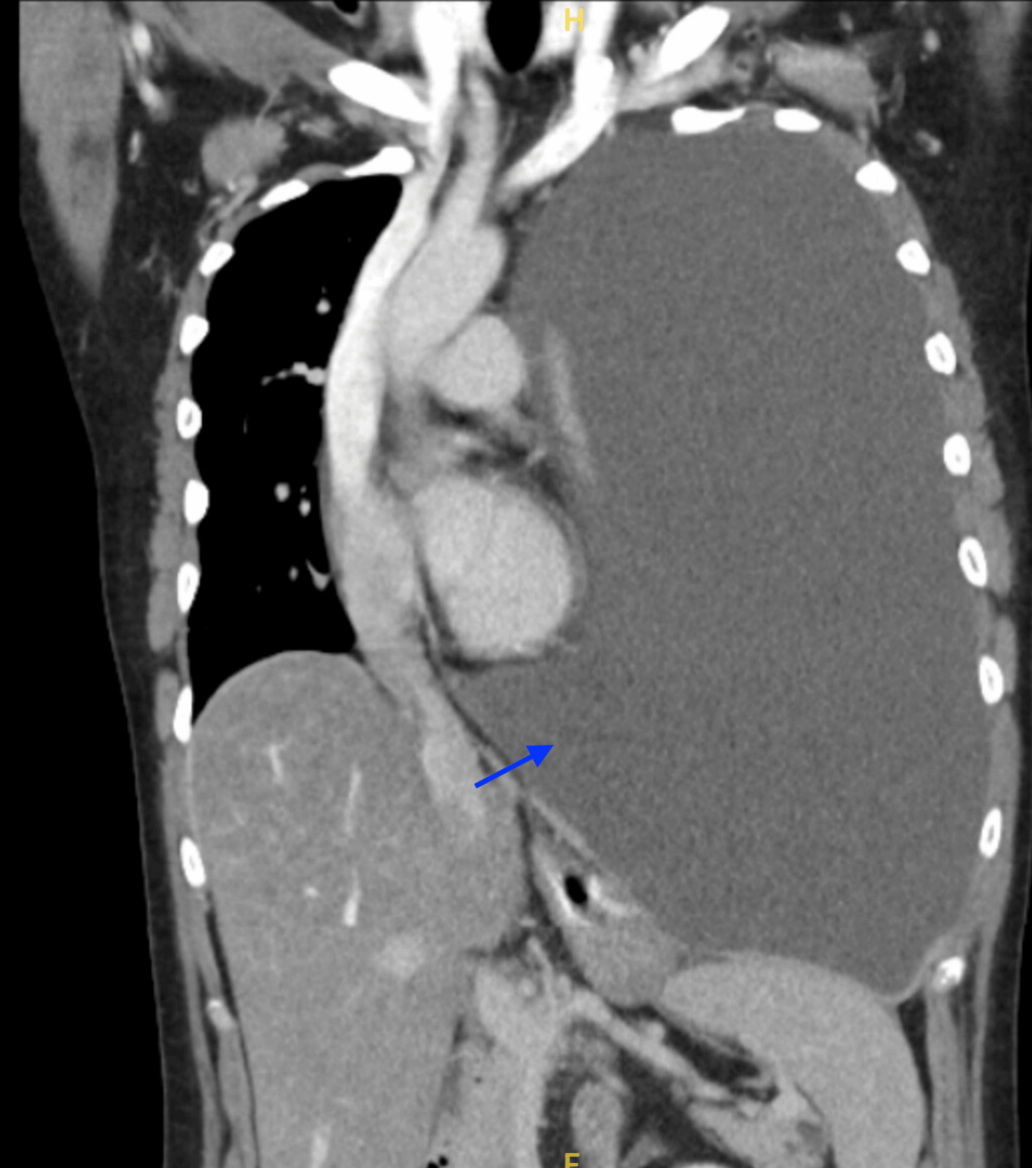
CT in coronal view, blue arrow pointing to a massive left pleural effusion occupying the entire hemithorax with total atelectasis of the left lung

Contrast-enhanced computed tomography (CT) of the thorax revealed a massive left pleural effusion occupying the entire hemithorax with total atelectasis of the left lung, inferior displacement of the diaphragm, and rightward mediastinal shift (Figure 1). Cardiothoracic surgery (CVTS) was consulted early due to the size of the effusion and associated mass effect.

A diagnostic and therapeutic thoracentesis drained 1 L of thick brown fluid, after which a left-sided pigtail chest tube was placed.

Pleural fluid analysis demonstrated brown cloudy fluid with pH 7.72, glucose at 146 mg/dL, total protein at 3.3 g/dL, albumin at 1.4 g/dL, lactate dehydrogenase at 606 U/L, white blood cell count at 299 cells/mm³ with 69% monocytes, 30% lymphocytes, and 1% granulocytes, triglycerides at 44 mg/dL, and amylase exceeded 1500 U/L (reference: <150 U/L). Serum lactate dehydrogenase (LDH) was not available; however, serum total protein was 6.0 g/dL, supporting classification as an exudative effusion by Light’s criteria.

The patient subsequently underwent ERCP, revealing a nondilated main pancreatic duct with distal contrast extravasation, demonstrating ductal disruption with fistulous communication into the thoracic cavity. A sphincterotomy was performed and an 8.5 Fr×5 cm transpapillary plastic pancreatic duct stent was placed.

**Figure 2:**
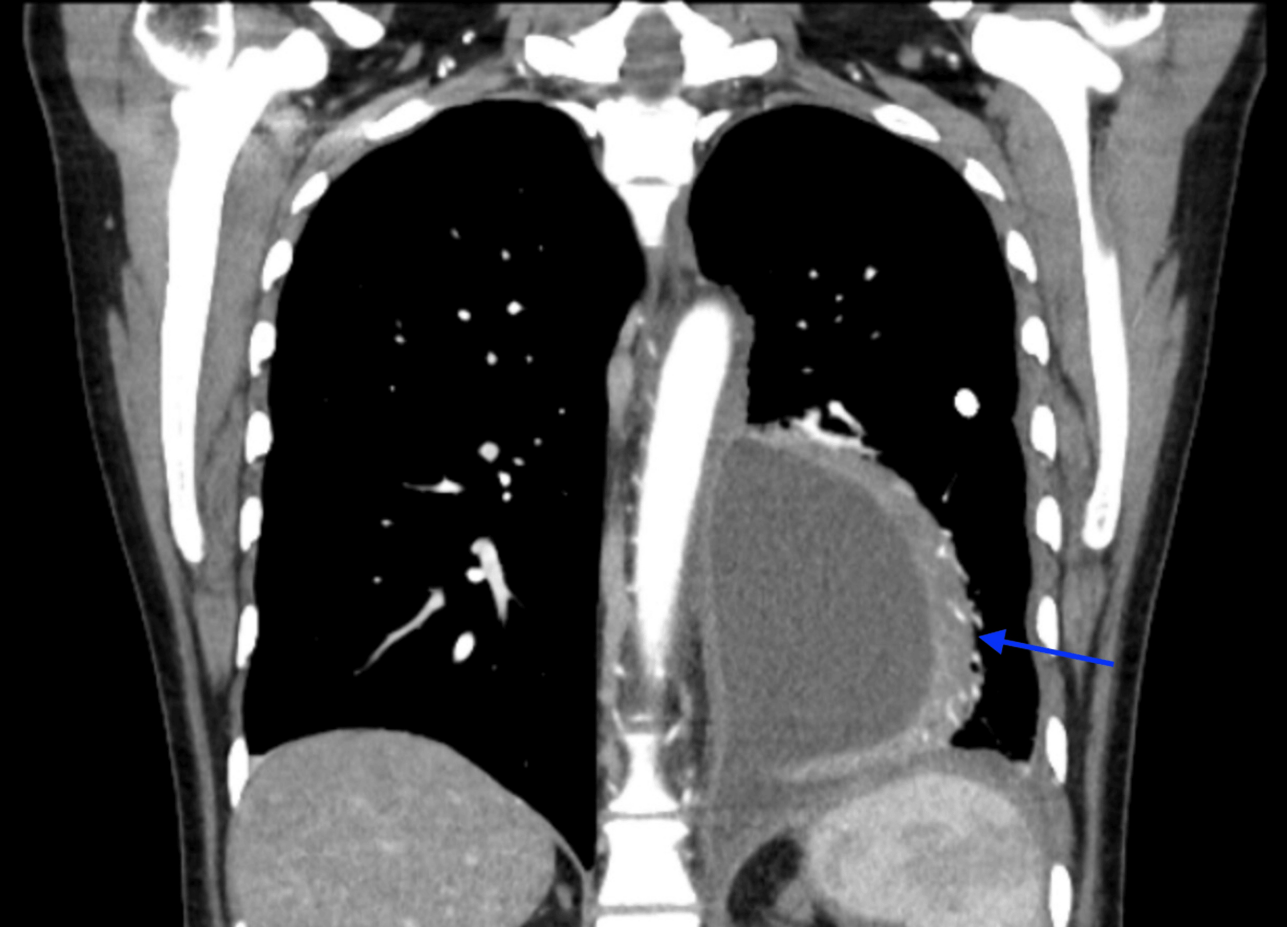
CT in coronal view, blue arrow pointing to rim-enhancing left lower lobe pulmonary abscess/empyema

**Figure 3:**
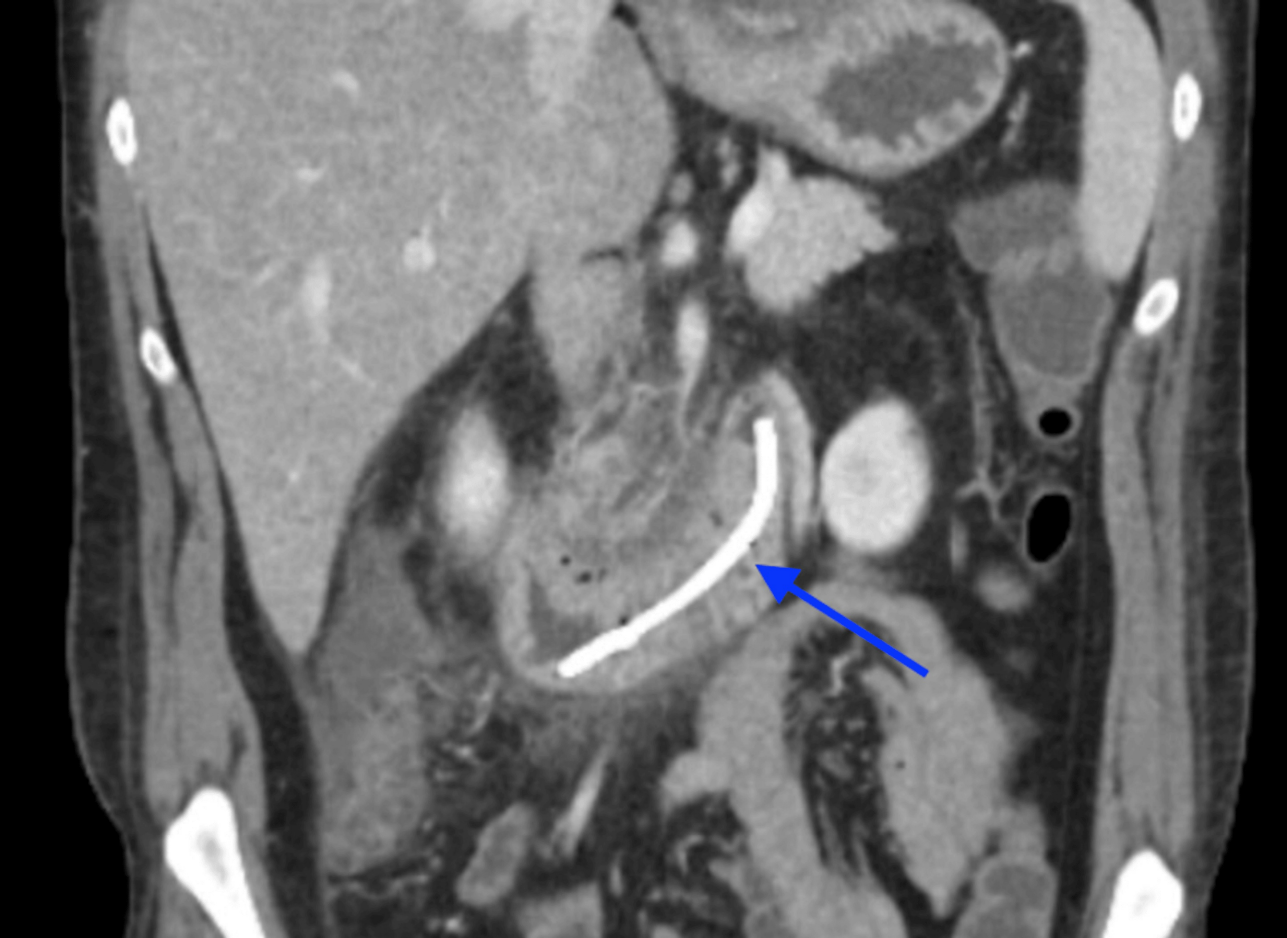
CT in coronal view, blue arrow pointing to a migrated pancreatic duct stent located within the duodenum

Within 24 hours of ERCP, she developed a fever of 39.4°C and leukocytosis with white blood cell count (WBC) increased to 17×10⁹/L (reference: 4.0-11.0×10⁹/L), prompting initiation of broad-spectrum antibiotics. A repeat CT of the chest revealed a rim-enhancing left lower lobe pulmonary abscess/empyema (Figure 2), multiple pancreatic pseudocysts, including one tracking through the diaphragm into the thoracic cavity, a migrated pancreatic duct stent located within the duodenum (Figure 3), and malposition of the existing chest tube outside the main pleural collection. CVTS re-evaluated the patient and recommended repositioning, and the malpositioned chest tube was subsequently removed. Interventional radiology placed an additional chest tube into the dominant loculated pleural collection for definitive drainage.

Pleural fluid cultures later demonstrated heavy growth of *Serratia liquefaciens* and *Pseudomonas putida*, confirming superinfection of the pancreaticopleural effusion. Broad-spectrum antimicrobial therapy was initiated with vancomycin and piperacillin-tazobactam, followed by escalation to meropenem and fluconazole. After culture-directed identification of *S. liquefaciens* and *P. putida*, therapy was transitioned to cefepime for approximately three weeks. At discharge, the patient was prescribed oral levofloxacin 750 mg daily for 20 days.

Due to stent migration, gastroenterology (GI) performed a repeat ERCP and placed a plastic stent in the ventral pancreatic duct with plans to remove it in two months. The patient’s oxygenation requirement decreased, leukocytosis downtrended, serial imaging showed successful resolution of pleural effusion, and the patient was discharged home.

The patient underwent initial chest tube placement on hospital day 1, ERCP with pancreatic duct stenting on hospital day 3, repeat ERCP with stent replacement on hospital day 6 following migration, and chest tube removal after radiographic improvement.

## Discussion

Most PPF cases present with recurrent unilateral pleural effusions but lack extensive thoracic pathology [4-6]. In contrast, this patient developed complete lung atelectasis causing mediastinal shift, empyema, pulmonary abscess, and a transdiaphragmatic pseudocyst, findings of which are rarely described in combination [7,8].

While abdominal pain may be present in some cases, PPF often manifests primarily with respiratory symptoms. Our patient’s absence of abdominal complaints is consistent with prior studies showing that thoracic manifestations can predominate and delay diagnosis [4,6]. Magnetic resonance cholangiopancreatography is often sufficient for identifying a fistulous tract [5]. In this case, ERCP was both diagnostic by demonstrating distal ductal contrast extravasation with fistulous communication into the pleura, as well as therapeutic by enabling decompression, in line with current management recommendations [1,3,9].

Most cases improve after pancreatic duct stenting alone [1,3,9]. This case was complicated by stent migration, polymicrobial infection, and extensive thoracic complications, yet the patient recovered without surgical intervention. Rapid stent migration may occur in the setting of a nondilated pancreatic duct, distal ductal disruption, short stent length, or incomplete bridging of the disruption site. Persistent ductal pressure gradients may also contribute to early displacement [9].

Similar high-severity cases in the literature more commonly proceed to thoracotomy, video-assisted thoracoscopic surgery, or pancreatic resection [7,10]. In this case, surgical intervention was considered, with video-assisted thoracoscopic surgery reserved pending repeat CT evaluation. Because repeat CT imaging demonstrated improvement in pleural opacification following drainage, operative intervention was deferred [5,10].

This case reinforces that even advanced PPF with complex thoracic manifestations can respond to endoscopic management when adequate drainage and ERCP-based ductal decompression are achieved.

## Conclusions

Severe PPF can present with extensive thoracic pathology, including lung collapse, empyema, and transdiaphragmatic pseudocyst formation. Markedly elevated pleural amylase should prompt rapid evaluation for pancreatic duct injury, particularly in patients with alcohol use. This case demonstrates that even advanced PPF with complications can be successfully managed with endoscopic therapy and drainage without surgical intervention. Early recognition remains critical, as delayed diagnosis may lead to progressive respiratory compromise and infectious complications. Multidisciplinary coordination among gastroenterology, interventional radiology, and thoracic surgery was essential to achieving source control and recovery. This case further supports the growing role of endoscopic management as a first-line approach even in complex presentations.
